# Generation of a Novel Oncolytic Vaccinia Virus Using the IHD-W Strain

**DOI:** 10.1089/hum.2020.050

**Published:** 2021-05-17

**Authors:** Jaeil Shin, Soon-Oh Hong, Minju Kim, Hyesun Lee, Hwanjun Choi, Joonsung Kim, Jieun Hong, Hyesoo Kang, Eunjin Lee, Soondong Lee, Byoungjae Kong, Minjung Kim, Heonsik Choi, Sujeong Kim

**Affiliations:** Institute of BioInnovation Research, Kolon Life Science, Seoul, Republic of Korea.

**Keywords:** oncolytic virus, vaccinia, IHD-W strain

## Abstract

Oncolytic viruses are promising cancer therapies due to their selective killing of tumor cells and ability to stimulate the host immune system. As an oncolytic virus platform, vaccinia virus has unique advantages, including rapid replication, a broad range of host targets, and a large capacity for transgene incorporation. In this study, we developed a novel oncolytic vaccinia virus with high potency and a favorable safety profile. We began with the International Health Department-White (IHD-W) strain, which had the strongest cytotoxicity against tumor cells among the four vaccinia virus strains tested. Next, several candidate viruses were constructed by deleting three viral genes (*C11R*, *K3L*, and *J2R*) in various combinations, and their efficacy and safety were compared. The virus ultimately selected, named KLS-3010, exhibited strong antitumor activity against broad targets *in vitro* and *in vivo*. Furthermore, KLS-3010 showed a favorable safety profile in mice, as determined by the biodistribution and body weight change. More promisingly, KLS-3010 was able to shift the tumor microenvironment to a proinflammatory state, as evidenced by an increase in activated lymphocytes after KLS-3010 administration, suggesting that this strain may elicit an oncolytic virus-mediated immune response. The KLS-3010 strain thus represents a promising platform for the further development of oncolytic virus-based cancer therapies.

## Introduction

Cancer is the second leading cause of death globally and was responsible for more than 9 million deaths in 2018.^1^ Recently, anticancer drugs such as immune checkpoint inhibitors that are intended to enhance host antitumor immunity have shown clinical benefit and changed the direction of cancer drug development. Oncolytic viruses are promising anticancer agents, with advantages such as direct tumor killing and immune stimulation. Various types of oncolytic viruses are being developed and tested in clinical trials worldwide, including the U.S. Food and Drug Administration (FDA)-approved Imlygic. However, the discovery and establishment of safer and more effective oncolytic viruses are still a top priority in this field.

Vaccinia virus is one of the most promising candidate oncolytic viruses due to advantages such as a favorable safety profile with smallpox vaccination history, a large capacity to harbor foreign genes,^2^ and an ability to infect a broad spectrum of host cells.^3^ Currently, several oncolytic vaccinia viruses based on the Western Reserve (WR),^4^ Wyeth,^5^ and Lister^6^ strains are in the preclinical and the clinical stages of development. Although different strains share the common benefits of vaccinia viruses, certain properties that are closely related to target cell cytotoxicity, including syncytia formation and the production of extracellular enveloped viruses (EEVs), differ among the strains.

In this study, we focused on the International Health Department-White (IHD-W) strain of vaccinia virus, which forms syncytia after host cell infection^7–9^ and has higher EEV production than other vaccinia strains.^10^ Multinucleated cell formation by viral fusion proteins may provide clinical benefits such as increased immunogenic cell death, which generates tumor-derived neoantigens and activates the host immune response against tumor cells,^11^ and/or increased virus spread from the initial site of infection.^12^ Virus spread can also be accelerated through production of EEVs.

Most oncolytic viruses, including vaccinia virus, are genetically engineered to attenuate their virulence and to selectively replicate in cancer cells but not healthy cells. Virus replication capability in the infected host cells depends on how efficiently the virus is able to use the host cell machinery. Vaccinia viruses with deletions of the genes encoding thymidine kinase (TK, *J2R*) and/or vaccinia growth factor (VGF, *C11R*) showed selective replication in cancer cells and reduced pathogenicity compared with the wild-type viruses^13–15^ and are already being used in clinical trials.

In addition to these two genes, we identified other candidate genes that may be involved in the regulation of cellular antiviral responses. The *K3L* gene product of vaccinia virus acts as a competitive inhibitor of the double-stranded RNA-dependent protein kinase (PKR) in infected host cells to prevent apoptotic cell death.^16^ However, in cancer cells, the PKR pathway is dysfunctional, and the vaccinia virus is able to continue replicating without a shortage of host cell resources even in the absence of the *K3L* gene. For example, the *K3L* gene product is required for virus replication in baby hamster kidney fibroblasts (BHK cells), but is dispensable in HeLa cells.^17^ Based on this evidence, we removed the *K3L* gene in addition to the well-known *J2R* and *C11R* genes from the vaccinia virus genome to improve safety.

Several oncolytic virus candidates were constructed by deletion of these three viral genes (*C11R*, *K3L*, and *J2R*) in various combinations, and *in vitro* and *in vivo* screenings were performed to investigate the safety and efficacy of these constructs. The construct lacking all three viral genes, which was named KLS-3010, showed high cancer-selective cytotoxicity against broad targets and was also safe in animals. Furthermore, administration of KLS-3010 in a syngeneic mouse tumor model induced a host immune response within the tumor environment. Collectively, these results suggest that the attenuated KLS-3010 vaccinia virus derived from the IHD-W strain represents an improved therapeutic modality with the ability to induce host antitumor immune responses as well as to selectively and efficiently lyse cancer cells.

## Materials and Methods

### Cell culture

The Hep3B, SKOV-3, U-118MG, U-87MG, FaDu, MCF-7, T-47D, DU145, A549, B16F10, LLC1, CT26.WT, HeLa, and NHBE (normal human bronchial epithelial) cell lines were purchased from the American Type Culture Collection (ATCC, VA). The HCT116, SW620, HT-1080, AsPC-1, MIA PaCa-2, and JC cell lines were purchased from the Korea Cell Line Bank (Seoul, Korea). A2780 and Vero cells were purchased from the European Collection of Authenticated Cell Cultures (ECACC, Salisbury, United Kingdom) and the Korean Ministry of Food and Drug Safety (Chungcheongbuk-do, Korea), respectively. All cells were cultured in an incubator at 37°C with 5% CO_2_ in distinct culture media (Supplementary Table 1) supplemented with fetal bovine serum (FBS) (SH30084.03; GE Healthcare Life Sciences, PA), antibiotic/antimycotic (A/A; 15240112), l-glutamine (25030164), and human insulin (12585014) (all from Thermo Fisher Scientific, MA).

### Plasmids

Shuttle plasmids to remove the single-viral genes *C11R* (C), *K3L* (K), or *J2R* (J) from the wild-type IHD-W vaccinia virus genome by homologous recombination were constructed based on the pSP72 vector (P2191; Promega, WI). Approximately 1 kb of both flanking regions of the target genes bearing reporter gene(s) (LacZ [240071-52; Agilent, CA] for C, DsRed2 [632404; Agilent] for K, and eGFP [6080-1; Clontech, CA] and Gpt [derived from DH5] for J) was cloned into the vectors. Final constructs were verified by enzyme mapping and sequence analysis.

### Viruses

The panel of wild-type vaccinia virus strains used in this study was purchased from ATCC (IHD-W [VR-1441], Lister [VR-1549], WR [VR-1354], and Wyeth [VR-1536]). All recombinant viruses were generated from the wild-type IHD-W strain.

For the generation of recombinant viruses, HeLa cells were seeded in six-well plates (3506; Corning, NY) 1 day before virus infection in Minimum Essential Media (MEM) supplemented with 10% FBS, 1% A/A, and 2 mM l-glutamine. The medium was replaced with the infection medium, which contained 2% FBS instead of 10%, and cells were infected with virus (either wild-type or single- or double-gene-deleted IHD-W vaccinia virus) at an multiplicity of infection (MOI) of 0.05 TCID_50_/cell. At 15 min postinfection, cells were transfected with 2 μg of shuttle plasmid using jetPRIME (114-01; Polyplus, Illkirch, France) and incubated for 4 h. The FBS concentration was then changed to 5%. After 48 h of culture, the medium was removed and cells were collected in 0.5 mL of infection medium. The crude virus was obtained by freezing and thawing three times.

For the isolation of single-virus clones, HeLa cells plated in six-well plates in infection medium were infected with crude virus and incubated for 2 h. Then, the medium was removed, and cells were overlaid with prewarmed 1% agarose (50100; Lonza, Basel, Switzerland) in RPMI (LM204-50; Welgene; Gyeongangbuk-do, Korea) supplemented with 2% FBS, 1% A/A, and 2 mM l-glutamine and cultured for 72 h to allow plaque formation. Plaques of target gene-deleted and reporter gene-inserted recombinant viruses were selected by visual observation. Selected viruses were amplified by infection of HeLa cells in T175 flasks (159910; Nunc, Roskilde, Denmark) for 48 h. Cells were harvested by scraping, and viruses were collected by sequential freezing and thawing followed by removal of cell debris by centrifugation.

For *in vivo* assays, viruses were further clarified by ultracentrifugation over a sucrose cushion (36% w/v)^18^ followed by resuspension of the viral pellet in 30 mM Tris-Cl, pH 9.0. The titers of all the viruses were determined by TCID_50_ assay on Vero cells, as described elsewhere,^19^ before use in *in vitro* and *in vivo* assays. Briefly, 10-fold serial dilutions of the viruses were prepared in culture medium before the infection of Vero cells in 96-well plates. At 4 days postinfection, the cytopathic effects were observed by optical microscopy, and the titers were calculated using the Spearman/Karber formula.

### *In vitro* cytotoxicity assays

For the vaccinia virus strain selection, human cancer cells (A549, SW620, MIA PaCa-2, and T-47D) were seeded in 96-well plates and infected with wild-type vaccinia virus strains (IHD-W, Lister, WR, and Wyeth) at MOIs of 0.1 TCID_50_/cell. Cell viability was determined 3 days postinfection using Cell Counting Kit-8 (CCK-8) assay (CK04; Dojindo, MD) and normalized to the level of noninfected control cells.

To test the cytotoxicity of KLS-3010 against the various human cancer cell lines listed in Supplementary Table 1, cells were seeded in 96-well plates and infected with KLS-3010 at an MOI of 0.5 TCID_50_/cell. Cell viability was measured at day 3–5 postinfection as described above and normalized to the level of noninfected cells.

For the comparison of the selective cytotoxicity of candidate viruses in cancer cell lines (SW620, T-47D, and A549) compared with normal primary cells (NHBE), cells were seeded in 96-well plates and infected with candidate viruses at MOIs of 0.001–0.1 (cancer cells) or 0.001–1 (NHBE cells). The cytotoxicity of each candidate virus was expressed as the median effective dose (ED_50_) and calculated by the linear interpolation method, as follows:

$$ED_{50}=fx_{2}+\left(1-f\right)x_{1},$$

where *x*_1_ and *x*_2_ represent the MOIs resulting in just greater than or less than 50% cell viability, respectively, while f is obtained from the formula below:

$$f=\frac{a}{\left(a+b\right)},$$

where *a* = [cell viability (%) just above 50%] − 50% while, *b* = 50% − [cell viability (%) just below 50%].

### Syncytia formation assays

Four wild-type virus strains (IHD-W, WR, Wyeth, and Lister) were tested for syncytia formation in the T-47D human breast cancer cell line. T-47D cells were seeded on coverslips and placed in six-well plates (Corning) in culture medium (RPMI supplemented with 10% FBS, 1% A/A, 2 mM l-glutamine, and 0.2 units/mL recombinant human insulin).

For virus infection, the culture medium was replaced with infection medium, which contains 2% FBS instead of 10%, and viruses were added at an MOI of 0.01 TCID_50_/cell. After 2 h of infection, the medium was replaced with fresh infection medium to discard unattached viruses, and cells were further incubated at 37°C in a 5% CO_2_ incubator for 40 h. The coverslips were transferred to six-well plates and washed with 1 mL of phosphate-buffered saline (PBS), then cells were fixed in 4% formaldehyde (163-20145; Fujifilm Wako Pure Chemical Corporation, Osaka, Japan) for 15 min. After washing with PBS, cells were permeabilized with 1 mL of 0.5% Triton X-100 dissolved in PBS for 10 min. Then, cells were treated with 5% bovine serum albumin (A2153; Sigma, MO) in PBS (blocking buffer) for 1 h to block nonspecific interactions.

To visualize actin in the cytosol, the cells were incubated with a primary antibody targeting β-actin (1:200, A1978; Sigma) in blocking buffer for 24 h at 4°C followed by incubation with an Alexa Fluor 594-conjugated donkey anti-mouse IgG secondary antibody (1:500, A-21203; Thermo Fisher Scientific) for 1 h at room temperature. Nuclei were directly stained with 4′,6-diamidino-2-phenylindole (DAPI, D9542; Sigma) diluted 1:500 in blocking buffer for 1 h at room temperature. Optical and fluorescent images were captured by microscopy (Axiovert 200; Carl Zeiss, Oberkochen, Germany) at a 400 × magnification.

### EEV production assay

HeLa cells on a six-well plate were infected with four wild-type vaccinia virus strains (IHD-W, WR, Wyeth, and Lister) at MOIs of 0.1. After 1 h of infection, the medium was removed and the cells were washed twice with DPBS, and fresh infection medium (MEM, 2% FBS, and 1% A/A) was added. Supernatants and cells were harvested separately 24 h after infection. Supernatants containing extracellular viruses were collected by centrifugation at 1,000 rpm for 5 min, and intracellular viruses were harvested from the cell pellet after three cycles of freezing/thawing. Infectious viruses in each fraction were quantified by the TCID_50_ assay.

### Animal experiments

Male BALB/c AnNCrj-nu nude mice between 5 and 6 weeks of age were purchased from Charles River Laboratories (Yokohama, Japan). Male BALB/c AnNHsd mice and female C57BL/6N mice were purchased from Koatech (Gyeonggi-do, Korea). Mice were housed in the animal facility at Kolon Life Science (Seoul, Korea). All animal experiments were approved by the Institutional Animal Care and Use Committee (IACUC) of Kolon Life Science (Approval Nos. KLS-IACUC-2015-46, 2015-48, 2017-151, 2019-169, 2019-172, and 2019-174).

### *In vivo* tumor models

For the xenograft model, 5 × 10^6^ SW620 cells were subcutaneously implanted on the right flanks of 6-week-old male BALB/c AnNCrj-nu nude mice. For syngeneic models, murine cancer cells were subcutaneously implanted on the right flanks of 6-week-old female C57BL/6N mice (5 × 10^5^ B16F10 or 1 × 10^6^ LLC1 cells) or 6-week-old male BALB/c mice (1 × 10^6^ CT26.WT or 5 × 10^6^ JC cells). Mice were randomized into study groups (*n* = 4–6), and when the tumor mass reached an average volume of 70–100 mm^3^ (7 days after cell inoculation), mice were injected with 50 μL of PBS or candidate viruses.

### *In vivo* antitumor efficacy

For the selection of candidate viruses, PBS or candidate viruses at a dose of 5 × 10^6^ TCID_50_ were administered intratumorally (i.t.) to mice bearing SW620 xenografts (*n* = 6 mice/group). For *in vivo* efficacy testing against various tumors, mice bearing CT26.WT, LLC1, or B16F10 cells (*n* = 6 mice/group) received PBS or KLS-3010 i.t. at doses of 1 × 10^8^, 1 × 10^8^, and 1 × 10^6^ TCID_50_, respectively. Tumor volumes and body weights were measured twice a week until the end of the observation period or until euthanasia of the mice. Mice were sacrificed when tumors reached 2,000 mm^3^.

### *In vivo* safety

The safety of select candidate viruses (J, CJ, CK, and CKJ) was determined *in vivo* in mice bearing SW620 xenografts. Viruses were administered i.t. at a dose of 5 × 10^6^ TCID_50_, and net body weight was calculated by subtracting the tumor mass (1 mm^3^ = 1 mg) from the total body weight. Tumor volume (V) was calculated by the formula below:

$$V=\frac{length\times width^{2}}{2}$$

Mice that were treated with the final two candidates (CJ and CKJ) were observed for up to 49 days to evaluate long-term mortality.

The safety profile of KLS-3010 under systemic exposure was assessed in 6-week-old immunocompetent BALB/c male mice (*n* = 6 mice/group). KLS-3010 and wild-type IHD-W virues were administered intravenously (i.v.) at a dose of 1 × 10^7^ TCID_50_. KLS-3010 was also injected i.v. at various doses (1 × 10^7^, 1 × 10^8^, and 1 × 10^9^ TCID_50_). For all studies, PBS was injected as a control. The clinical signs and body weights were observed once a day until day 35.

### *In vivo* biodistribution in mice

B16F10 tumor-bearing mice (*n* = 6 mice/group) were injected with PBS or KLS-3010 i.t. at a dose of 2 × 10^8^ TCID_50_. At 6 and 120 h after administration, mice were sacrificed and various tissues, including the blood, brain, lungs, liver, heart, kidneys, ovaries, and injection site were harvested. Total DNA was extracted from each sample using the DNeasy Blood and Tissue Kit (69506; Qiagen, Venlo, Netherlands) according to the manufacturer's protocol. The virus genome copy number was quantified by quantitative polymerase chain reaction (qPCR) (7900HT; Applied Biosystems) targeting the *A4L* gene of vaccinia virus. The primers and probe were purchased from Macrogen (Seoul, Korea) (forward primer: 5′-CGTTTGTTTCGGCCTGAAG-3′, reverse primer: 5′-CAGGAGCAGCATCTCAACAAAA-3′, and probe: 5′'-FAM-ACTCGACATGAGATCCTTAAGGGCCA-TAMRA-3′).

All qPCRs were carried out in a mixture of 300 ng template DNA (extracted gDNA or the *A4L* plasmid, as a standard), 900 nM each primer, 250 nM probe, and 10 μL TaqMan master mix (4304437; Applied Biosystems) in a final volume of 20 μL. All reactions included negative controls and were run in triplicate. Data were analyzed with SDS software, v.2.4 (Applied Biosystems).

### Flow cytometry

For the analysis of immune cell populations, LLC1 tumor-bearing mice (*n* = 5 mice/group) were treated with PBS or KLS-3010 at a dose of 1 × 10^6^ TCID_50_. At day 5 post-treatment, mice were sacrificed to harvest tumors. The tumors were minced into small fragments and then incubated with a mixture of 0.15 mg/mL DNase type I (D5025-375KU) and 1 mg/mL collagenase type IV (C5138-1G; Sigma) in Dulbecco's modified Eagle's medium at 37°C for 30 min. Erythrocytes were removed using red blood cell lysis buffer (420301; Biolegend, CA).

The dissociated cells were stained with the following antibodies: CD3-BV510 (563024; BD Biosciences, CA), CD4-PE-Cyanine7 (47-0041-82), CD4-APC-eFlouor780 (25-0041-82), CD8a-APC-eFlouor780 (45-0081-82), CD25-APC-eFlouor780 (47-0251-82), FoxP3-Percp-Cyanine5.5 (45-5773-82), and IFN-γ-PE-Cyanine7 (25-7311-82). Fc block (14-0161-85) and normal mouse serum (24-5544-94) were used to prevent nonspecific antibody binding before surface antigen staining and intracellular staining, respectively.

Dead cells were excluded with a fixable viability dye (65-0864-14). Intracellular staining was performed using the Foxp3/Transcription Factor Staining Buffer Set (00-5523-00) according to the manufacturer's protocol. All reagents listed above except the CD3-BV510 were purchased from Thermo Fisher Scientific. Immunofluorescence was measured on an FACS Canto II and analyzed using FACSDiva software (BD Biosciences).

### Statistical analyses

Statistical analyses were carried out using SigmaPlot, version 13. Data are expressed as the mean ± standard error of the mean. Statistical significance was determined by one-way ANOVA (analysis of variants) with the Student/Newman/Keuls method for multiple comparisons or Student's *t*-tests for comparisons between two groups. Survival curves were generated by the Kaplan/Meier method. The log rank test was used to statistically compare two groups. *p*-Values were considered to be statistically significant at <0.05.

## Results

### Selection of the vaccinia virus strain based on cytotoxicity

**Figure 1.**
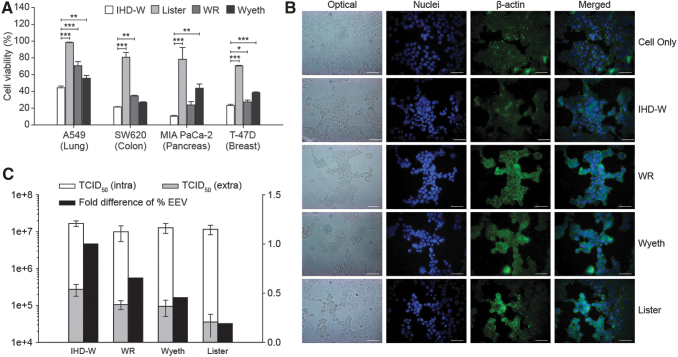
Comparison of cancer cell cytotoxicity and other characteristics among vaccinia virus strains. **(A)** Four human cancer cell lines (A549, SW620, MIA PcCa-2, and T-47D) were infected with four wild-type vaccinia virus strains (IHD-W, WR, Wyeth, and Lister) at an MOI of 0.1 TCID_50_/cell. Three days after infection, cell viability was measured by CCK-8 assay. Cell viability results are expressed as the percentage of viable virus-infected cells relative to noninfected cells (100% viability). All conditions were run in triplicate, and the data represent the mean ± standard error. *p < 0.05, **p < 0.01, and ***p < 0.001. **(B)** T-47D cancer cells were infected with four wild-type vaccinia virus strains at an MOI of 0.01 TCID_50_/cell. Cell morphology was observed at 42 h postinfection by optical and fluorescence microscopy to determine syncytia formation. In the fluorescent images, nuclei (*blue*) were labeled with DAPI, and β-actin (*green*) was labeled by the fluorophore Alexa Fluor 594. **(C)** HeLa cells were infected with the four wild-type vaccinia strains at an MOI of 0.1 TCID_50_/cell, and the culture media were changed at 1 h postinfection to remove unbound viruses. Intracellular viruses (*white bar*) and extracellular viruses (*gray bar*) in the cell culture supernatant were collected separately at 24 h postinfection and the quantity of infectious viruses was determined by the TCID_50_ assay. The proportion of released virus was normalized to that of the IHD-W strain (*black bar*). All conditions were run in triplicate, and the data represent the mean ± standard error. CCK-8, Cell Counting Kit-8; DAPI, 4′,6-diamidino-2-phenylindole; IHD-W, International Health Department-White; MOI, multiplicity of infection; WR, Western Reserve.

The most cytotoxic vaccinia virus strain was selected for further engineering by comparing the viability of human cancer cell lines upon infection with various vaccinia virus strains. Four vaccinia virus strains (IHD-W, WR, Wyeth, and Lister) were used to infect various human cancer cell lines (A549, SW620, MIA PaCa-2, and T-47D) at an MOI of 0.1 mean tissue culture infectious dose (TCID_50_)/cell, and the cell viability was measured by CCK-8 assay at day 3 postinfection. Among the tested vaccinia virus strains, IHD-W exhibited the highest cytotoxicity against all cancer cell lines screened (Fig. 1A), and it was therefore selected as the parental strain for the generation of oncolytic viruses.

### Syncytia formation upon infection with the IHD-W strain

To understand the mechanisms responsible for the improved cytotoxicity of the IHD-W stain compared with the other vaccinia virus strains, we evaluated syncytia formation, which potentially contributes to target cell death. Generally, vaccinia virus and other Poxviridae family members are known not to cause cell-to-cell fusion to generate multinucleated cells, also called syncytia. The complex between two vaccinia virus proteins, hemagglutinin (*A56R*) and serine protease inhibitor-3 (*K2L*), on the infected cell surface prevents fusion with neighboring infected and uninfected cells.

However, the IHD-W strain expresses a truncated version of *A56R* lacking the transmembrane domain and is able to form syncytia upon infection of host cells.^20,21^ The truncation of *A56R* specifically in the IHD-W strain was confirmed by gene sequence analysis (Supplementary Fig. S1).

To confirm that syncytia formation was induced by IHD-W, the four vaccinia virus strains were used to infect T-47D human cancer cells, and the cell-to-cell fusion was observed by optical and fluorescence microscopy at 42 h postinfection. Infection with the WR, Wyeth, and Lister strains resulted in a very similar altered cell morphology, with individual and compartmentalized round cells containing a single nucleus and no fused cells. However, infection with the IHD-W strain induced the formation of large single cells containing multiple nuclei surrounded by a single cytosol (Fig. 1B).

### Higher EEV production by the IHD-W strain

Another characteristic of vaccinia virus that may affect its cytotoxicity is the production of extracellular enveloped virus (EEV) by infected host cells. Only a few *Orthopoxvirus* strains, including rabbitpox, International Health Department-Japan (IHD-J), and IHD-W, are known to express a variant of the *A34R* protein with a mutation (K151E) in the lectin-like domain (Supplementary Fig. S2), and expression of this mutant *A34R* protein directly influences the production of EEV.^22^ Production of intracellular and extracellular progeny viruses in HeLa cells infected with four wild-type vaccinia virus strains was determined by the TCID_50_ assay. All strains produced a major population of progeny viruses inside the cells, while IHD-W released a comparably high level of progeny viruses from cells (Fig. 1C).

This supports the idea that dissemination of IHD-W is superior during host cell infection, resulting in increased cytotoxicity.

### Enhanced safety through viral gene deletion

The IHD-W strain was selected for development as an oncolytic virus based on its strong cancer cell killing activity and other useful characteristics. However, removal of viral genes from the wild-type IHD-W strain is required to promote cancer cell-selective replication and reduce the virulence of the strain. For this purpose, the viral genes *C11R*, *K3L*, and *J2R* were deleted from the wild-type IHD-W vaccinia virus in various combinations. A total of seven candidate viruses with single (C, K, or J)-, double (CK, CJ, or KJ)-, or triple (CKJ)-gene deletions were generated by homologous recombination^23^ and the genetic identity was confirmed by PCR and nucleotide sequencing of the modified regions (data not shown).

**Figure 2.**
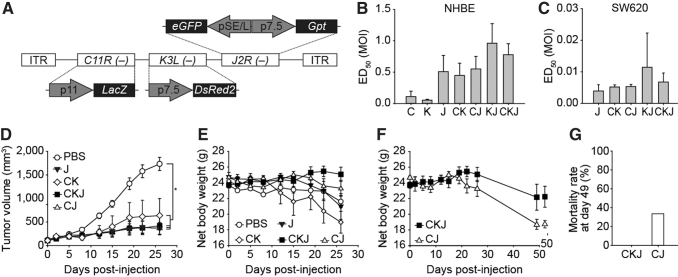
Selection of the final oncolytic virus construct based on *in vitro* and *in vivo* efficacy and safety. **(A)** Three viral genes (*C11R*, *K3L*, and *J2R*) were selected, and seven candidate viruses were generated by removing various combinations of these three viral genes. For the selection of genetically modified virus, reporter genes (LacZ, DsRed2, eGFP, and Gpt) were inserted into the viral genomic loci indicated by the *dashed lines*. ITR, inverted terminal repeat. **(B, C)** The median effective dose (ED_50_) of various candidate viruses was calculated at day 5 after infection of normal primary (NHBE) cells **(B)** or the cancer cell line SW620 **(C)**. All conditions were run in triplicate and the average ED_50_ was obtained from one to three independent experiments. **(D–G)** *In vivo* safety and efficacy tests of select candidate viruses were performed in a mouse xenograft tumor model. SW620 cells were injected subcutaneously into BALB/c nude mice, and candidate viruses were administered i.t. at a dose of 5 × 106 TCID_50_. The antitumor efficacy **(D)** and net body weight **(E)** for the four candidate viruses were measured for 26 days. The observation period was extended to 49 days for the final two candidates (CJ and CKJ), to compare the net body weight **(F)** and the mortality rate **(G)** over a longer period of time. Data are shown as the mean ± standard error (*n* = 6 mice/group). i.t., intratumorally; NHBE, normal human bronchial epithelial.

The reporter genes *lacZ*, *DsRed2*, and *EGFP/gpt* were inserted into the loci of *C11R*, *K3L*, and *J2R*, respectively, and used for the isolation of target gene-deleted virus clones (Fig. 2A).

The cancer cell-specific cytotoxicity of the seven candidates was determined using both primary cells (NHBE cells) and human cancer cell lines (SW620, A549, and T-47D) by CCK-8 assay. Each virus was used to infect target cells at an MOI of 0.001–10 TCID_50_/cell to obtain the 50% effective dose (ED_50_), which indicates the concentration of infectious virus that results in 50% cell death. Two viruses with single-gene deletions (C and K) exhibited relatively strong cytotoxicity against NHBE cells compared with the other candidates, indicating less attenuation (Fig. 2B). One virus with a double-gene deletion (KJ), which had weak cytotoxicity against cancer cell lines, was also excluded (Fig. 2C and Supplementary Fig. S3). Based on *in vitro* cytotoxicity assays, four candidates (J, CK, CJ, and CKJ) were further tested in *in vivo* efficacy and safety studies to select the final construct.

To analyze the antitumor efficacy along with the safety of the selected candidates, nude BALB/c mice were implanted subcutaneously with SW620 cells, and 7 days later, the mice were treated i.t. with PBS or the four candidate viruses at a concentration of 5 × 10^6^ TCID_50_. The tumor volumes and body weights of the mice were measured until 26 days after injection. On day 26, the study was terminated because the average tumor size of the PBS group reached 2,000 mm^3^. Among the candidates, the CJ and CKJ viruses showed higher antitumor efficacy than the J and CK viruses (Fig. 2D), and did not significantly affect the body weight (Fig. 2E).

To further examine the safety profile, extended observation up to day 49 was conducted in the CJ- and CKJ-injected mice. This analysis revealed that the triple-gene-deleted virus (CKJ) had a better safety profile than the double-gene-deleted virus (CJ), as it resulted in less weight loss (Fig. 2F) and no mortality (Fig. 2G). Based on these results, CKJ was selected as the final construct, and it is referred to as KLS-3010 hereafter. The *in vitro* cancer cell selectivity and *in vivo* antitumor efficacy of KLS-3010 were better than those of the well-known oncolytic virus platform vvDD^15^ (Supplementary Fig. S4).

### Evaluation of the *in vivo* safety and biodistribution of KLS-3010

**Figure 3.**
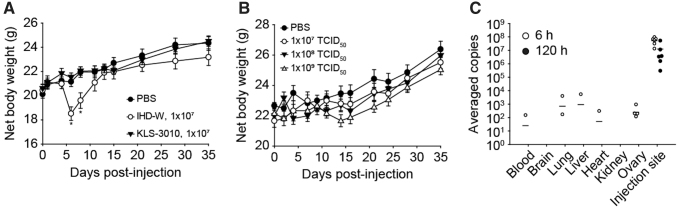
*In vivo* safety profile and biodistribution of KLS-3010 in normal and tumor-bearing mice, respectively. **(A)** KLS-3010 or wild-type IHD-W virus was injected intravenously into BALB/c mice at a dose of 1 × 107 TCID_50_, and PBS was used as a control. Body weight was monitored at the indicated time points (*n* = 6 mice/group). *p < 0.05. **(B)** KLS-3010 was injected intravenously into BALB/c mice at doses of 1 × 107, 1 × 108, and 1 × 109 TCID_50_. Body weight was measured at the indicated time points. Data are shown as the mean ± standard error (*n* = 6 mice/group). **(C)** Female C57BL/6N were implanted subcutaneously with B16F10 (melanoma) cells followed by intratumoral injection of KLS-3010 at a dose of 2 × 108 TCID_50_. Copies of the viral genome of KLS-3010 in various tissues (blood, brain, lungs, liver, heart, kidneys, and ovaries) and the tumor mass were quantified by qPCR at 6 (*open circle*) and 120 h (*closed circle*) postinjection. Averaged copies are indicated as *solid line*. Data are shown as mean ± standard error (*n* = 6 mice/group). PBS, phosphate-buffered saline; qPCR, quantitative polymerase chain reaction.

The safety of KLS-3010 was further evaluated by the measurement of the body weight after administration of KLS-3010 to healthy, nontumor-bearing mice. KLS-3010 and the wild-type IHD-W strain were i.v. administered to BALB/c mice at a dose of 1 × 10^7^ TCID_50_. PBS was used as a vehicle control. There was no body weight loss in mice injected with KLS-3010, but significant weight loss was observed at day 6 in the mice injected with wild-type IHD-W, suggesting that KLS-3010 has an improved safety profile compared with the wild-type virus (Fig. 3A).

To evaluate the safety of systemic exposure to KLS-3010 at a higher dose, KLS-3010 was injected i.v. at three dosing levels of 1 × 10^7^–1 × 10^9^ TCID_50_, and body weight was monitored over time. None of the groups showed a significant change in body weight, and there was no mortality until 35 days after injection (Fig. 3B). These results indicate that systemic exposure of KLS-3010 was tolerable up to a dose of 1 × 10^9^ TCID_50_.

The biodistribution of KLS-3010 was evaluated in immunocompetent tumor-bearing mice to determine the viral persistence and dissemination from the initial injection site, the tumor mass. KLS-3010 was administered i.t. at a dose of 2 × 10^8^ TCID_50_ to C57BL/6N mice bearing B16F10 tumors. Various tissues, including the blood, brain, lungs, liver, heart, kidneys, ovaries, and tumor, were collected at 6 and 120 h after injection.

Genomic DNA (gDNA) was extracted, and the amount of KLS-3010 genetic material was quantified by qPCR targeting the conserved vaccinia virus gene *A4L*. At 6 h postinjection, copies of the viral genome were detected in all organs except the brain, and the amount of viral genetic material at the injection site was several logs higher than at the other sites. The number of copies of viral genome at all sites except the injection site was below the lower limit of quantification at 120 h postinjection. At the injection site, considerable amounts of viral genomes were detected at 120 hours post-injection, although the amount of viral genomes was gradually decreased after the peak level reached at 24 hours post-injection (Fig. 3C and Supplementary Fig. S5).

### *In vitro* and *in vivo* antitumor efficacy of KLS-3010

**Figure 4.**
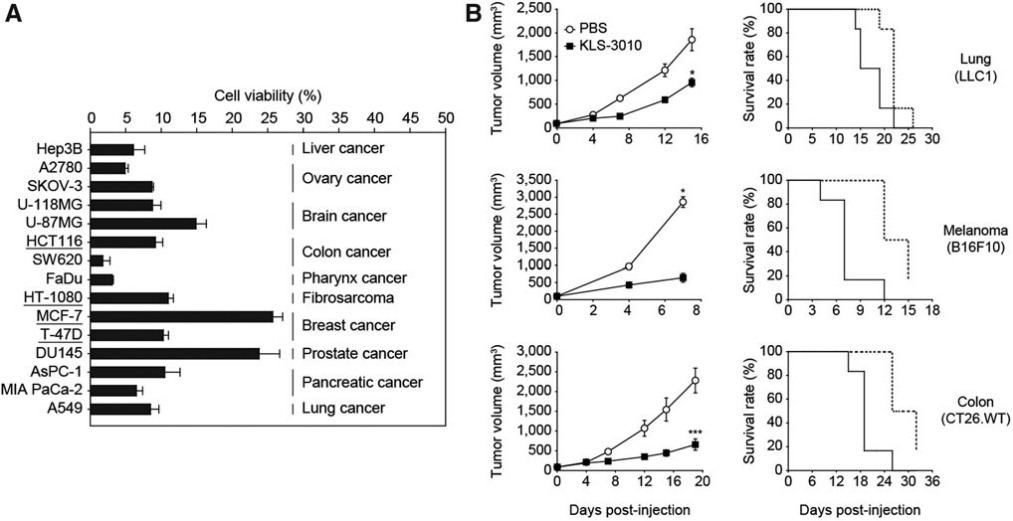
*In vitro* and *in vivo* oncolytic effect of KLS-3010 on various cancer cell lines. **(A)** The *in vitro* cytotoxicity of KLS-3010 was measured against various human cancer cell lines. Cells were infected with KLS-3010 at an MOI of 0.5 TCID_50_/cell, and cell viability was measured by CCK-8 assay at day 5 postinfection and normalized to the viability of noninfected cells. Certain cells were incubated for 3 (HCT116 and HT-1080) or 4 (MCF-7 and T-47D) days postinfection to measure cell viability. Data are shown as mean ± standard error. **(B)** The antitumor efficacy and survival upon KLS-3010 treatment were evaluated in immunocompetent murine tumor models. Mice were inoculated with LLC1 (lung), B16F10 (melanoma), or CT26.WT (colon) tumor cells and treated with KLS-3010 i.t. The administered doses were 1 × 106 TCID_50_/mouse in the B16F10 tumor model and 1 × 108 TCID_50_/mouse in the LLC1 and CT26.WT models. Data are shown as mean ± standard error (*n* = 6 mice/group). *p < 0.05 and ***p < 0.001.

The antitumor activity of KLS-3010 was evaluated using various human cancer cell lines. A total of 15 human cancer cell lines derived from 10 different organs were infected with KLS-3010 *in vitro* and the cell viability was evaluated. The cancer cell lines used for the viability study include Hep3B (liver), A2780 and SKOV-3 (ovary), U-118MG and U-87MG (brain), HCT116 and SW620 (colon), FaDu (pharynx), HT-1080 (fibrosarcoma), MCF-7 and T-47D (breast), DU145 (prostate), AsPC-1 and MIA PaCa-2 (pancreatic), and A549 (lung). All cell lines showed less than 25% viability after 3–5 days of infection at an MOI of 0.5, indicating that KLS-3010 had a broad range of target cancer cell tropism (Fig. 4A).

Next, the *in vivo* efficacy of KLS-3010 was evaluated in immunocompetent mice bearing LLC1 (lung cancer), B16F10 (melanoma), or CT26.WT (colon cancer) tumors. Single intratumoral administration of KLS-3010 led to a significant reduction in tumor growth and a considerable increase in the survival rate compared with PBS treatment in each of the three tumor models (Fig. 4B). None of the mice showed negative clinical signs such as body weight loss, skin lesions, or mobility issues as a result of the virus treatment (data not shown). Before testing *in vivo*, the susceptibility of murine cell lines to KLS-3010 was confirmed *in vitro* (Supplementary Fig. S6).

### Immune activation in the tumor upon intratumoral injection of KLS-3010

**Figure 5.**
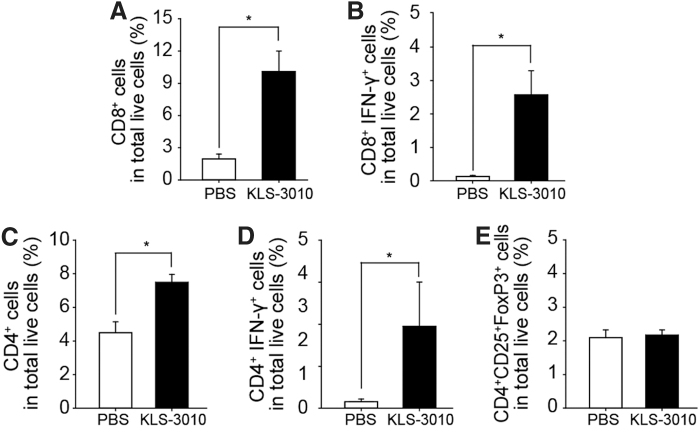
Increased tumor-infiltrating T cell populations upon treatment with KLS-3010. Tumor-infiltrating T cells were analyzed on day 5 postadministration. The percentages of CD8^+^ **(A)**, CD8^+^ IFN-γ^+^ **(B)**, CD4^+^ **(C)**, CD4^+^ IFN-γ^+^ **(D)**, and regulatory **(E)** T cells were determined by flow cytometry. Data are shown as mean ± standard error (*n* = 5 mice/group). *p < 0.05.

To determine whether KLS-3010 enhances immune responses in the tumor, mice bearing LLC1 tumors were injected i.t. with KLS-3010 or PBS. Five days after KLS-3010 administration, the percentages of CD8^+^ T cells and CD4^+^ T cells in the tumors were measured (Fig. 5). CD8^+^ T cells and CD4^+^ T cells were significantly increased in KLS-3010-injected mice compared with the control group (Fig. 5A and C). Interferon (IFN)-γ^+^ CD8^+^ T cells and INF-γ^+^ CD4^+^ T cells were also substantially increased by KLS-3010 administration (Fig. 5B and D). By contrast, there was no difference in the percentage of regulatory T cells (T_reg_) between PBS- and KLS-3010-treated mice (Fig. 5E). These results suggest that KLS-3010 treatment increases tumor-infiltrating T cells and augments cytotoxic T cell activity in the tumor, although it remains to be further seen whether this increased T cells are injected virus- or tumor-specific.

## Discussion

Effective host cell killing and induction of immune responses are key factors to the success of oncolytic virotherapy in the clinic.

To further this goal, we constructed a novel oncolytic virus based on the IHD-W strain of vaccinia virus, which had the strongest cytotoxicity among the strains tested in this study (Fig. 1A). The IHD-W strain is a laboratory passage clone of IHD-J, which is a derivative of the New York City Board of Health (NYCBH) vaccine strain,^24^ and induces syncytia formation in infected host cells because it is hemagglutinin (*A56R*)-negative.^25^ Furthermore, a single amino acid mutation (K151E) in the lectin-like domain of the *A34R* protein of WR or substitution of the IHD-W *A34R* gene into the WR strain both increase production of EEVs,^22,26^ which mediate the long-range dissemination of viruses.^27^ It is also reported that partial deletion of the B5R gene increases EEV productivity, which can be applied to improve the efficacy of oncolytic vaccinia viruses.^28^

In this study, we confirmed that the IHD-W strain harbors these distinct characteristics compared with other strains (Fig. 1). These characteristics of the IHD-W strain may potentially increase its cytotoxicity by enabling the virus to spread rapidly within the tumor microenvironment due to cell-to-cell fusion^29^ and the dissemination of progeny virions across neighboring host cells. More promisingly, syncytia formation may bring the added clinical benefit of enhanced antitumor immune responses, as the death of large, multinucleated cells is generally immunogenic.^30^ These properties of the wild-type IHD-W strain were maintained or even improved in KLS-3010 (Supplementary Fig. S7), which lacks three viral genes, indicating that these genes are not directly related to strain benefit.

Safety, along with a high tumor cell killing ability, is a major consideration for the development of oncolytic viruses.

We generated several candidates through deletion of three viral genes (*C11R*, *K3L*, and *J2R*) in various combinations and determined the final construct by stepwise screening. First, viruses that could kill normal cells were excluded, because they could cause toxicity in clinical applications. In that regard, two single-gene-deleted viruses (C and K viruses) were excluded. Second, viruses that had weak cancer cell killing activity were also excluded. One double-gene-deleted virus (KJ) was also excluded due to its weak ability to kill cancer cell lines, including SW620, A549, and T-47D.

After the screening, four candidates (J, CK, CJ, and CKJ) remained, and their efficacy was tested *in vivo*. All four candidates could control tumor growth in immunocompromised SW620 tumor-bearing mice. However, the body weights of J- and CK-treated animals decreased significantly compared with the other groups. With extended observation up to day 49, CKJ-treated mice remained alive, while significant body weight loss and a 33% death rate (two out of six, Fig. 2G) were observed in the CJ-treated group. Tumor volume did not differ between the CJ- and CKJ-treated groups during the entire observation period (Supplementary Fig. S8).

The difference between CJ and CKJ (KLS-3010) was the presence or absence of the *K3L* gene, which is known to inhibit the PKR pathway in infected host cells and prevent induction of antiviral defense mechanisms. It has been reported that vaccinia virus lacking the *K3L* gene is less pathogenic than the wild-type virus, and shows less systemic dissemination when injected intratracheally in C57BL/6N mice, although the mechanism is vague.^31^ Deletion of the *K3L* gene leaves the host defense system active and also seems to trap the virus locally, which likely improves the *in vivo* safety of KLS-3010 without altering the antitumor efficacy.

Despite the *K3L* gene deletion, there was concern that production of EEVs by KLS-3010 could increase the spread of the virus from the injection site and cause treatment-related adverse effects. A biodistribution study of KLS-3010 in the B16F10 syngeneic mouse model indicated that KLS-3010 was mainly located at the injection site, and only a small amount of virus was detected in other organs, and only at early time points (6 h postinjection) (Fig. 3C). Considering that the replication cycle of vaccinia virus takes ∼6 h,^32^ disseminated viruses found at 6 h after injection would likely represent leakage from the injection site, not replicated progeny virus.

To evaluate the safety of KLS-3010 *in vivo*, KLS-3010 was injected into normal mice via the intravenous route, representing a worst-case scenario in which all of the intended intratumoral dose misses the tumor and enters into the systemic circulation. KLS-3010 was administrated to normal mice i.v. at doses up to the maximum feasible dose, 1 × 10^9^ TCID_50_. As shown in Fig. 3B, no considerable body weight change was observed in any group. These results indicate the favorable safety profile of KLS-3010 for systemic exposure at high doses.

One of the advantages of the vaccinia virus as an oncolytic virus platform is that it is capable of infecting and replicating in various types of target cells. *In vitro* cytotoxicity and *in vivo* antitumor efficacy experiments confirmed that KLS-3010 maintained the broad target tropism of the parental virus strain (Fig. 4A). These data suggest that KLS-3010 could be used clinically for several types of cancer.

Syncytia formation, which is a notable characteristic of the IHD-W strain, is likely to induce immunogenic cell death^33^ and consequently activate antitumor immune cells in the tumor microenvironment.^30^ Intratumoral administration of KLS-3010 in the LLC1 syngeneic mouse model increased the number of lymphocytes, including CD8^+^ and CD4^+^ T cells, in the tumor at 5 days after injection. Moreover, the percentage of T_reg_ (CD4^+^, CD25^+^, FoxP3^+^), which is associated with a poor prognosis,^34,35^ was not altered by KLS-3010, while effector T cells (T_eff_; CD8^+^ IFF-γ^+^, and CD4^+^ IFF-γ^+^) were significantly increased among tumor-infiltrating lymphocytes in KLS-3010-treated mice (Fig. 5).

Reprogramming the tumor microenvironment from “cold” (noninflamed) to “hot” (inflamed) would be a major benefit of oncolytic virus therapy, both as a monotherapy and also in combination with other standard therapies. Although globally approved immunotherapies have been successful at improving survival,^36^ patients with cold tumors fail to benefit from immunotherapy, and combination treatment with an oncolytic virus could help overcome this limitation.

Although the novel oncolytic virus KLS-3010 shows tumor selectivity, a favorable safety profile, and the potential to boost the host immune response to the tumor, it is possible that its antitumor activity could be further improved by adding other transgenes to its genome. In particular, our current work focuses on manipulation of KLS-3010 to express transgenes that boost host immune responses, with the goal that this further engineered oncolytic virus could be used to treat patients with less immunogenic tumors.

## Supplementary Material

Supplemental data

Supplemental data

Supplemental data

Supplemental data

Supplemental data

Supplemental data

Supplemental data

Supplemental data

Supplemental data
