# Supplementary material for: Generation of a Novel Oncolytic Vaccinia Virus Using the IHD-W Strain
Source: Hum Gene Ther. 2021 May 17;32(9-10):517–27. doi: 10.1089/hum.2020.050 (PMC8140350; doi:10.1089/hum.2020.050)

**Supplementary Table 1.** The culture media used for the indicated cell lines are as follows: MEM (11090099), RPMI 1640 (21870092) (Thermo Fisher Scientific), DMEM (Welgene, LM001-08, Gyeongangbuk-do, Korea), McCoy’s 5A (30-2007), and Airway Epithelial Cell Basal Medium (PCS-300-030) with Bronchial Epithelial Cell Growth Kit (PCS-300-040) (ATCC, VA, USA).


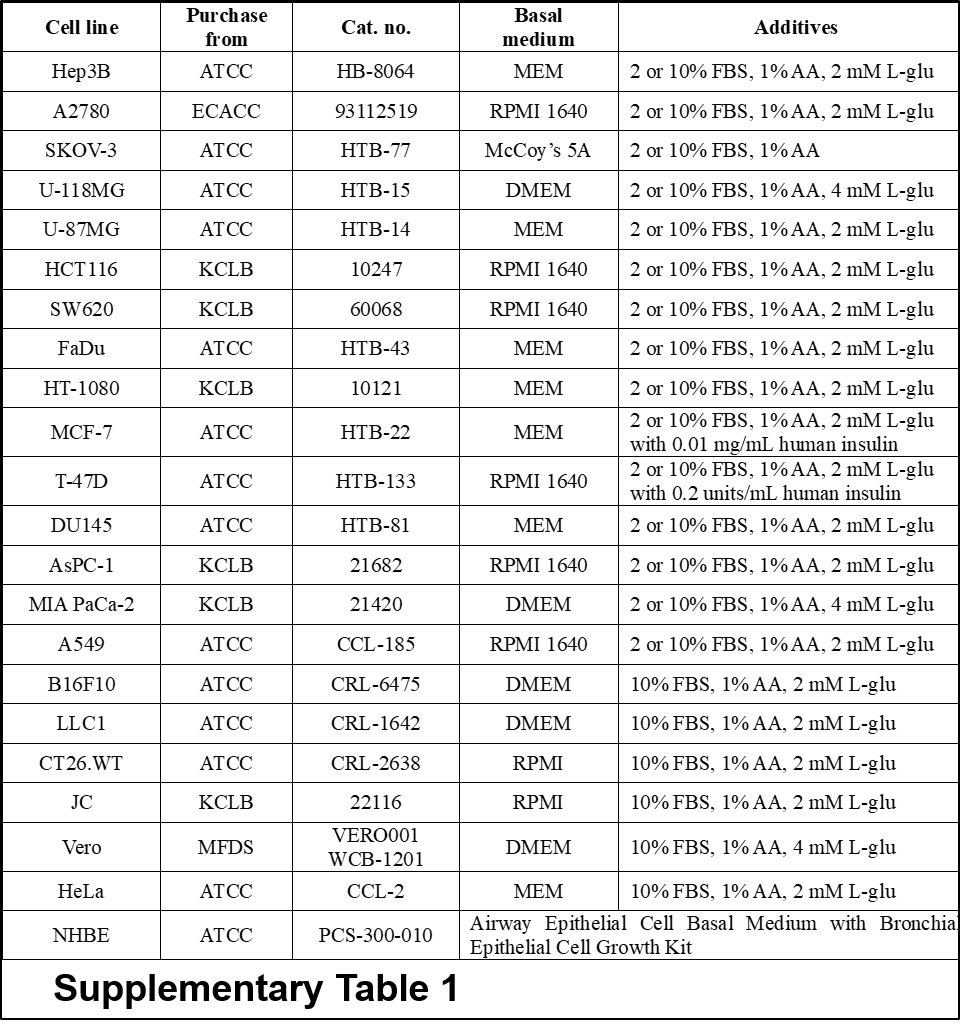

Supplement: Supplemental data [file Supp_TableS1.docx]
