# Supplementary material for: Generation of a Novel Oncolytic Vaccinia Virus Using the IHD-W Strain
Source: Hum Gene Ther. 2021 May 17;32(9-10):517–27. doi: 10.1089/hum.2020.050 (PMC8140350; doi:10.1089/hum.2020.050)

**Supplementary Figure S2.** Sequence alignment of the vaccinia virus A34R protein between the IHD-W (1), WR (2), and Lister (3) strains. Amino acids are indicated by the single letter code and the single amino acid mutation (K151E) of the IHD-W strain is depicted as a red square.


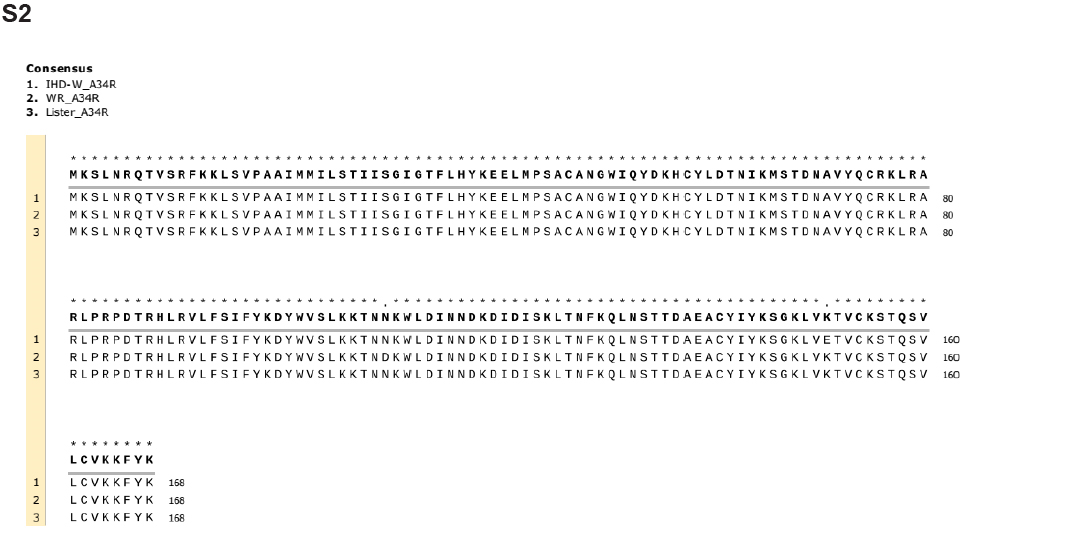

Supplement: Supplemental data [file Supp_FigS2.docx]
