# Supplementary material for: Generation of a Novel Oncolytic Vaccinia Virus Using the IHD-W Strain
Source: Hum Gene Ther. 2021 May 17;32(9-10):517–27. doi: 10.1089/hum.2020.050 (PMC8140350; doi:10.1089/hum.2020.050)

**Supplementary Figure S3.** The median effective dose (ED_50_) of various candidate viruses was calculated at Day 3 after infection of the cancer cell lines T-47D (open bar) and A549 (closed bar).


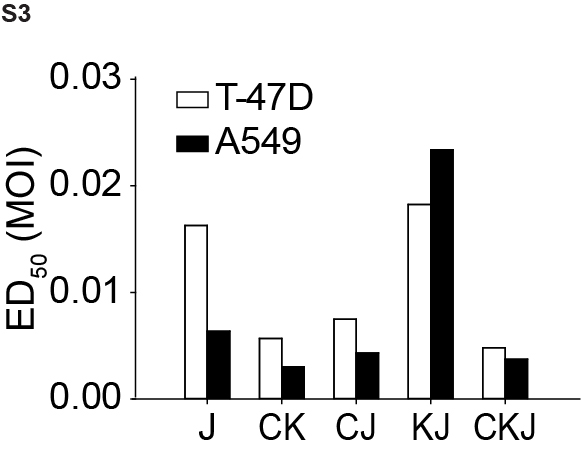

Supplement: Supplemental data [file Supp_FigS3.docx]
