# Supplementary material for: Generation of a Novel Oncolytic Vaccinia Virus Using the IHD-W Strain
Source: Hum Gene Ther. 2021 May 17;32(9-10):517–27. doi: 10.1089/hum.2020.050 (PMC8140350; doi:10.1089/hum.2020.050)

**Supplementary Figure S4.** Comparison of *in vitro* cancer cell selectivity and *in vivo* antitumor efficacy between vvDD and KLS-3010. (A) The median effective dose (ED_50_) of vvDD and KLS-3010 was calculated at Day 5 after infection of normal primary (NHBE) cells (left) or the cancer cell line SW620 (right). (B) *In* *vivo* efficacy and safety of vvDD and KLS-3010 were tested in a mouse xenograft tumor model. BALB/c nude mice were injected subcutaneously with SW620 cells and injected intratumorally with vvDD and KLS-3010 at a dose of 5 × 10^6^ TCID_50_. Tumor volume and body weight were measured for 26 days. Data are shown as mean ± standard error (n = 6 mice/group). *p < 0.05..


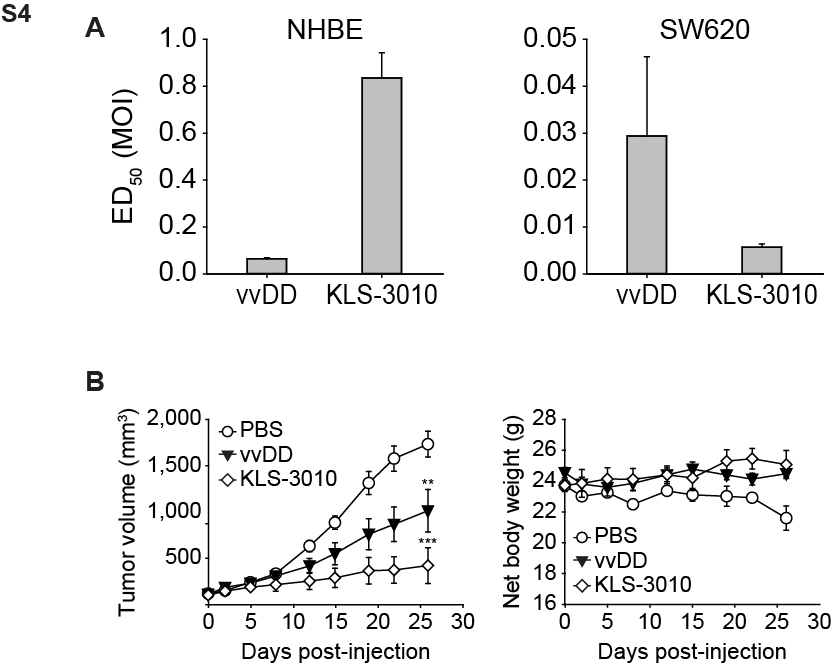

Supplement: Supplemental data [file Supp_FigS4.docx]
