# Supplementary material for: Generation of a Novel Oncolytic Vaccinia Virus Using the IHD-W Strain
Source: Hum Gene Ther. 2021 May 17;32(9-10):517–27. doi: 10.1089/hum.2020.050 (PMC8140350; doi:10.1089/hum.2020.050)

**Supplementary Figure S5.** Biodistribution of KLS-3010 in tumor-bearing mice. Female C57BL/6N mice were implanted subcutaneously with B16F10 (melanoma) cells and then intratumorally injected with KLS-3010 at a dose of 2 × 10^8^ TCID_50_. Copies of the KLS-3010 viral genome at the injection site (tumor) were quantified by qPCR from 6 hours to 5 days post-injection. The average number of copies is indicated in the bar graph. Data are shown as mean ± standard error (n = 6 mice/group).


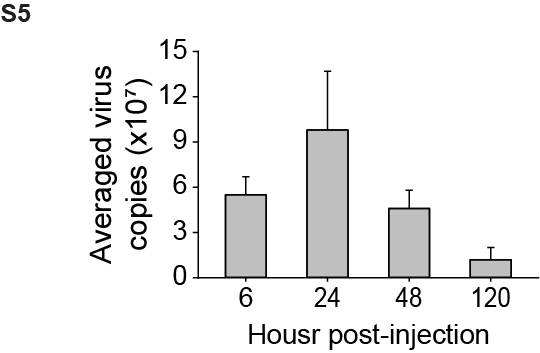

Supplement: Supplemental data [file Supp_FigS5.docx]
