# Supplementary material for: Generation of a Novel Oncolytic Vaccinia Virus Using the IHD-W Strain
Source: Hum Gene Ther. 2021 May 17;32(9-10):517–27. doi: 10.1089/hum.2020.050 (PMC8140350; doi:10.1089/hum.2020.050)

**Supplementary Figure S6.** Confirmation of KLS-3010 cytotoxicity in murine cell lines. Three murine cancer cell lines (LLC1, B16F10, and CT26.WT) were infected with KLS-3010 at an MOI of 0.01–10 TCID_50_/cell. At 3 days post-infection, the cell viability was measured by CCK-8 assay. Uninfected cells were used as controls (100% viability).


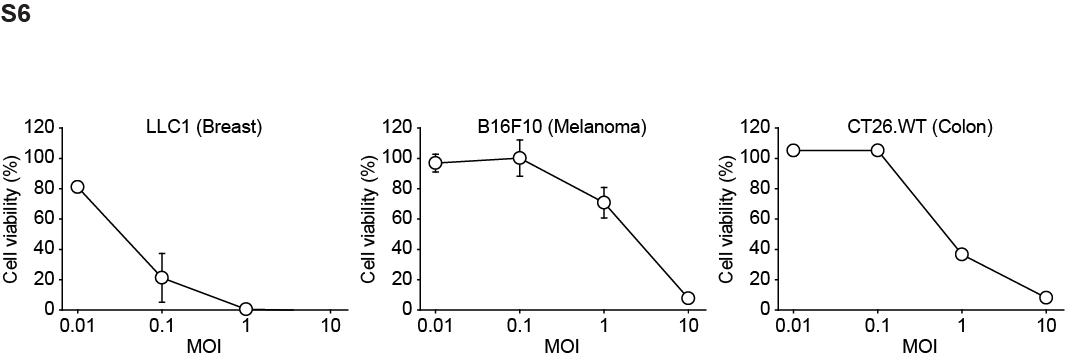

Supplement: Supplemental data [file Supp_FigS6.docx]
