# Supplementary material for: Generation of a Novel Oncolytic Vaccinia Virus Using the IHD-W Strain
Source: Hum Gene Ther. 2021 May 17;32(9-10):517–27. doi: 10.1089/hum.2020.050 (PMC8140350; doi:10.1089/hum.2020.050)

**Supplementary Figure S7.** (A) T-47D cancer cells were infected with the wild-type IHD-W vaccinia virus strain and KLS-3010 at an MOI of 0.01 TCID_50_/cell. Cell morphology was observed at 42 hours post-infection by optical and fluorescence microscopy to determine syncytia formation. In the fluorescence images, nuclei (blue) were labeled with DAPI, and β-actin (green) was labeled by the fluorophore Alexa Fluor 594. (B) Newly produced virus by HeLa cells infected with the wild-type IHD-W vaccinia strain and KLS-3010 was compared quantitatively. Viruses were infected at an MOI of 0.1 TCID_50_/cell and the culture media was changed at 1 hour post-infection to remove unbound virus. Intracellular virus (white bar) and virus in the cell culture supernatant (gray bar) were collected separately at 24 hours post-infection to quantify infectious virus by the TCID_50_ assay. The proportion of released virus in each strain was normalized to that of IHD-W (black bar). All conditions were run in triplicate, and the data represents the mean ± standard error.


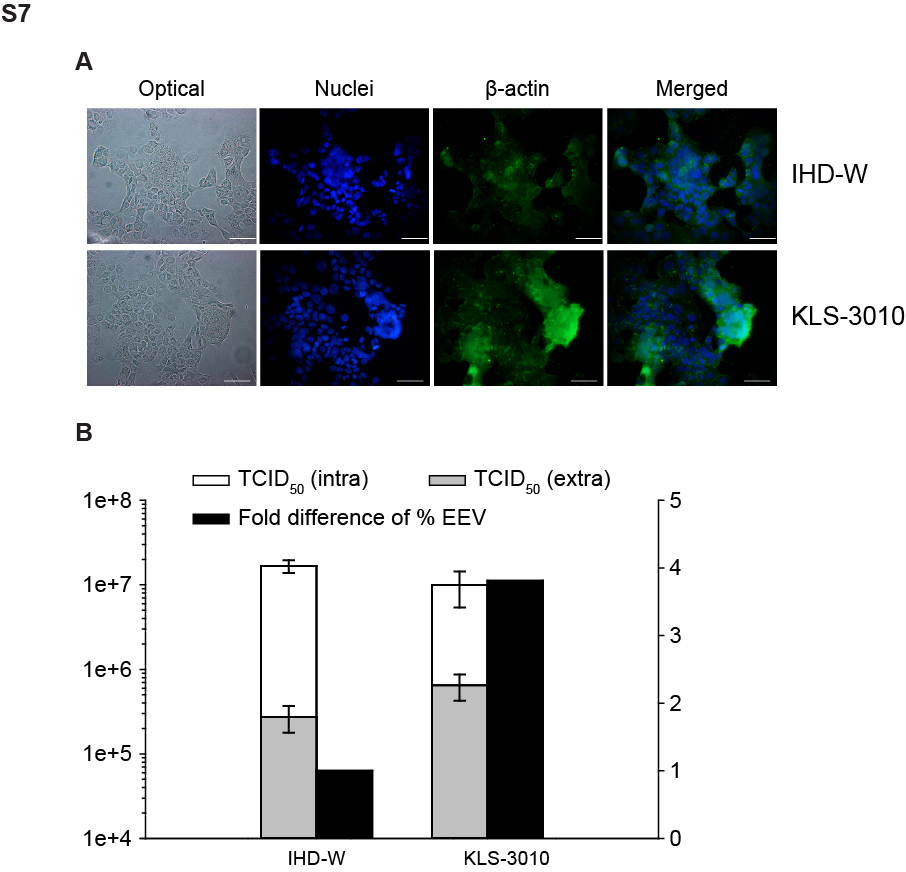

Supplement: Supplemental data [file Supp_FigS7.docx]
