# Supplementary material for: Generation of a Novel Oncolytic Vaccinia Virus Using the IHD-W Strain
Source: Hum Gene Ther. 2021 May 17;32(9-10):517–27. doi: 10.1089/hum.2020.050 (PMC8140350; doi:10.1089/hum.2020.050)

**Supplementary Figure S8.** Change in tumor volume after administration of CJ and CKJ viruses. BALB/c nude mice were injected subcutaneously with SW620 cells and injected intratumorally with CJ and CKJ viruses at a dose of 5 × 10^6^ TCID_50_. Tumor volume was measured for 49 days. During the experiment, two mice died, one at Day 36 and another at Day 49, after virus administration in the CJ virus-treated group. Data are shown as mean ± standard error (n = 6 mice/group). Tumor volume in the CJ-treated group at Day 49 was calculated with five mice because one mouse died at Day 36.


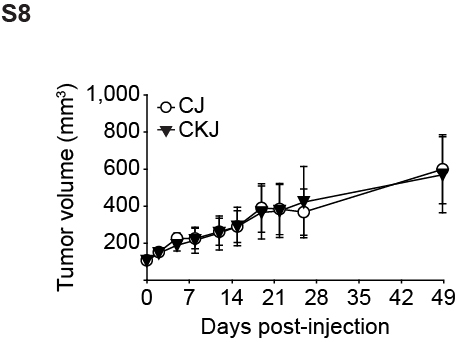

Supplement: Supplemental data [file Supp_FigS8.docx]
